# Prevalence and Cure of Primary Liver Cancer in Italy by Histologic Type

**DOI:** 10.3390/cancers18142318

**Published:** 2026-07-18

**Authors:** Lauro Bucchi, Stefano Ferretti, Federica Toffolutti, Ugo Fedeli, Silvia Mancini, Federica Zamagni, Fabiola Giudici, Laura Botta, Manuel Zorzi, Ettore Bidoli, Francesco Cuccaro, Cinzia Gasparotti, Adele Caldarella, Mario Fusco, Monica Lanzoni, Enrica Migliore, Antonella Puppo, Maria Michiara, Rossella Cavallo, Margherita Ferrante, Giuseppe Sampietro, Walter Mazzucco, Claudia Cirilli, Rosa Vattiato, Fabrizio Stracci, Linda Guarda, Federica Manzoni, William Mantovani, Giuseppe Cascone, Caterina Palmonari, Federica Targa, Lucia Mangone, Rocco Galasso, Antonio Porcheddu, Maria Teresa Pesce, Francesca Bella, Pietro Seghini, Anna Clara Fanetti, Pasquala Pinna, Stefano Guzzinati, Luigino Dal Maso, on behalf of the AIRTUM Working Group

**Affiliations:** 1 Emilia-Romagna Cancer Registry, Romagna Unit, IRCCS Istituto Romagnolo per lo Studio dei Tumori (IRST) Dino Amadori, 47014 Meldola, Italy; lauro.bucchi@irst.emr.it (L.B.); silvia.mancini@irst.emr.it (S.M.); federica.zamagni@irst.emr.it (F.Z.); rosa.vattiato@irst.emr.it (R.V.); 2 Emilia-Romagna Cancer Registry, Ferrara Unit, 44121 Ferrara, Italy; stefano.ferretti@unife.it (S.F.); c.palmonari@ausl.fe.it (C.P.); 3 University of Ferrara, 44121 Ferrara, Italy; 4 Cancer Epidemiology Unit, Centro di Riferimento Oncologico di Aviano (CRO) IRCCS, 33081 Aviano, Italy; federica.toffolutti@cro.it (F.T.); fabiola.giudici@cro.it (F.G.); bidolie@cro.it (E.B.); 5 Epidemiological Department, Azienda Zero, 35132 Padua, Italy; ugo.fedeli@azero.veneto.it (U.F.); manuel.zorzi@azero.veneto.it (M.Z.); 6 Evaluative Epidemiology Unit, Department of Epidemiology and Data Science, Fondazione IRCCS Istituto Nazionale Dei Tumori, 20133 Milan, Italy; laura.botta@istitutotumori.mi.it; 7 Agenzia Regionale Strategica per la Salute e il Sociale (AReSS) Puglia, UOC Epidemiologia e Care Intelligence, 70121 Bari, Italy; f.cuccaro@aress.regione.puglia.it; 8 Struttura Semplice Dipartimentale (SSD) Epidemiologia, ATS Brescia, 25124 Brescia, Italy; cinzia.gasparotti@ats-brescia.it; 9 Tuscany Cancer Registry, Clinical Epidemiology Unit, Institute for Cancer Research, Prevention and Clinical Network (ISPRO), 50139 Florence, Italy; a.caldarella@ispro.toscana.it; 10 UOSD Registro Tumori ASL Napoli 3 Sud, 80035 Naples, Italy; mariofusco2@virgilio.it; 11 Registro Tumori ATS Insubria (Provincia di Como e Varese), 21100 Varese, Italy; lanzonim@ats-insubria.it; 12 Piedmont Cancer Registry, CPO Piemonte and University of Turin, 10123 Torino, Italy; enrica.migliore@cpo.it; 13 Liguria Cancer Registry, IRCCS Ospedale Policlinico San Martino, 16132 Genova, Italy; antonella.puppo@hsanmartino.it; 14 Emilia-Romagna Cancer Registry, Parma Unit, Medical Oncology Unit, University Hospital of Parma, 43126 Parma, Italy; mariamichiara@gmail.com; 15 Cancer Registry ASL Salerno-Dipartimento di Prevenzione, 84129 Salerno, Italy; registrotumori@aslsalerno.it; 16 Registro Tumori Integrato di CT-ME-EN, UOC Igiene Ospedaliera, Azienda Ospedaliero-Universitaria Policlinico G. Rodolico-San Marco, 95123 Catania, Italy; segreteria.rti@policlinico.unict.it; 17 Servizio Epidemiologico Aziendale, ATS di Bergamo, 24121 Bergamo, Italy; 18 Clinical epidemiology and Cancer Registry Unit, Azienda Ospedaliera Universitaria Policlinico (AOUP) di Palermo, 90127 Palermo, Italy; walter.mazzucco@unipa.it; 19 Emilia-Romagna Cancer Registry, Modena Unit, Public Health Department, Local Health Authority, 41121 Modena, Italy; c.cirilli@ausl.mo.it; 20 Section of Public Health, Department of Medicine, University of Perugia; Registro Tumori dell’Umbria, Regione Umbria, 06128 Perugia, Italy; fabrizio.stracci@unipg.it; 21 SSC Osservatorio Epidemiologico, ATS Val Padana, 46100 Mantova, Italy; linda.guarda@ats-valpadana.it; 22 Cancer Registry of the Province of Pavia, Epidemiology Unit, Agency for Health Protection of Pavia, 27100 Pavia, Italy; federica_manzoni@ats-pavia.it; 23 Trento Province Cancer Registry, Unit of Clinical Epidemiology, 38123 Trento, Italy; william.mantovani@asuit.tn.it; 24 Azienda Sanitaria Provinciale di Ragusa, Dipartimento di Prevenzione, UOSD Registro Tumori, 97100 Ragusa, Italy; giuseppe.cascone@asp.rg.it; 25 South Tyrol Cancer Registry, Innovation, Research and Teaching Service (SABES-ASDAA), Lehrkrankenhaus der Paracelsus Medizinische Privatuniversität, 39100 Bolzano-Bozen, Italy; federica.targa@sabes.it; 26 Emilia-Romagna Cancer Registry, Reggio Emilia Unit, Epidemiology Unit, Azienda Unità Sanitaria Locale—IRCCS di Reggio Emilia, 42122 Reggio Emilia, Italy; lucia.mangone@ausl.re.it; 27 Epidemiology and Regional Cancer Registry Unit, IRCCS CROB, 85028 Rionero in Vulture, Italy; rocco.galasso@crob.it; 28 Nord Sardegna Cancer Registry, ASL, 07100 Sassari, Italy; antonio.porcheddu@aslsassari.it; 29 Environmental Risk Monitoring and Cancer Registry Unit, ASL Caserta, 81100 Caserta, Italy; direzione@registrotumoricaserta.it; 30 Siracusa Cancer Registry, Provincial Health Authority of Siracusa, 96100 Siracusa, Italy; francesca.bella@asp.sr.it; 31 Emilia-Romagna Cancer Registry, Piacenza Unit, Public Health Department, AUSL Piacenza, 29121 Piacenza, Italy; epidemiologiapc@ausl.pc.it; 32 Cancer Registry of ATS Montagna, Agenzia di Tutela della Salute della Montagna, 23100 Sondrio, Italy; ac.fanetti@ats-montagna.it; 33 Nuoro Cancer Registry, RT Nuoro, Servizio Igiene e Sanità Pubblica, ASL Nuoro, 08100 Nuoro, Italy; pasquala.pinna@aslnuoro.it

**Keywords:** liver cancer, hepatocellular carcinoma, intrahepatic cholangiocarcinoma, prevalence, cure fraction

## Abstract

Background/Objectives: In Italy, one- and five-year survival of primary liver cancer patients have increased in recent decades and were higher after hepatocellular carcinoma (HCC) than after intrahepatic cholangiocarcinoma (ICC). This study aimed to estimate the complete prevalence and cure indicators (long-term survival) after liver cancer diagnosis by histologic type. Methods: Data from 31 Italian cancer registries, covering 28,057,000 residents (47% of the population), were included. The complete prevalence was calculated in 2018. The mixture-cure model was used to estimate the cure fraction (CF, proportion of newly diagnosed patients with the same life expectancy as the general population), the cure prevalence (prevalent patients who will not die as a result of cancer) and the life expectancy of fatal cases (LEF). Results: In 2018, 31,723 Italians (53 per 100,000) lived after a liver cancer diagnosis. Among them, the cure prevalence was 23.3% for HCC and 40.3% for ICC. The CF remained <10% for cases diagnosed in recent decades. Higher and rising CF rates were observed between 2005 and 2015 only among patients with HCC aged <45 years (2% of all cases), with an increase from 13% to 22% in men and from 19% to 29% in women, and among patients aged 45–54 years (8% of all cases), with an increase from 7% to 15% in men and from 11% to 19% in women. The LEF for HCC in 2015 was 29 months for both sexes, an 8-month increase since 2005. Conclusions: No substantial advantage nor favourable trends in the CF was apparent for older patients with HCC as compared to those with ICC, with some progresses for the small age groups of <45 years. A clear-cut increase in CF of patients with HCC aged under 55 years occurred in the 2010s decade, with no changes for older patients. This was accompanied by an increase in the LEF. Although limited by a considerable proportion of liver cancers of unspecified histologic type, the cure indicators presented here may help monitor the long-term effects of therapeutic advances and of surveillance programmes for people with cirrhosis and HCV infection.

## Simple Summary

In Italy, short-term survival for primary liver cancer patients has improved in recent decades and is higher for patients with hepatocellular carcinoma (HCC) than for those with intrahepatic cholangiocarcinoma (ICC). To obtain further information on current national policies for liver cancer control, we estimated the complete prevalence and indicators of cure (long-term survival) by histologic type. In 2018, 53 per 100,000 Italians (*n *= 31,723) lived after a liver cancer diagnosis. Among them, the cure prevalence was 23.3% for HCC and 40.3% for ICC. Patients with HCC had a cure fraction <10% both among early and more recent cases. Greater and increasing cure fractions were observed in the small groups of younger patients. The life expectancy of uncured patients with HCC also displayed an increase. The cure indicators may help to determine the long-term effects of therapeutic advances and of surveillance programmes for people with cirrhosis and HCV infection.

## 1. Introduction

The vaccination against the hepatitis B virus (HBV) [1], the use of direct-acting antivirals (DAAs) against the hepatitis C virus (HCV) [2] and the adoption of lifestyle changes to prevent alcohol-related liver disease [3] and metabolic dysfunction-associated steatotic liver disease [4] were demonstrated to be the most effective means for reducing primary liver cancer morbidity and mortality [5]. However, early detection and effective treatment remain key components of a comprehensive strategy for the control of the disease. Imaging surveillance for chronic HCV infection and cirrhosis, to detect liver cancer at a more treatable tumour stage [6,7], and patient access to state-of-the-art medical services [2,8] are the cornerstones of the clinical management of the disease. Notably, there are 38 different surveillance systems in 27 European countries, and six of these countries have more than one system [9].

Over the last two decades, major advances in treatment have included the introduction of chinase inhibitors, multiple embolisation [10] and ablation techniques [11], loco-regional radiation therapies [12], minimally invasive surgery [13], and combination immunotherapies [13]. In addition, liver transplantation for hepatocellular carcinoma (HCC), the most common histologic type, has increasingly been used [14]. Liver transplantation offers a putatively curative treatment both for the cancer and the underlying chronic liver disease [15,16].

In Italy, despite decreasing incidence rates in both sexes [17], the risk of liver cancer is still one of the world’s highest, excluding Asia [18]. The country, however, also ranks high in patient survival on a global scale [19]. Between 2003 and 2017, both men and women experienced improvements in 1-, 2-, 5- and 10-year net survival (NS), as well as 5|1- and 5|2-year conditional NS after HCC [17]. The uptrend in NS after the less frequent and less curable intrahepatic cholangiocarcinoma (ICC) was moderate and significant only for women [17,20].

Another clinically valuable feature of HCC in Italy is the high proportion attributable to HCV infection, that is, 50–60% versus a global average of 19% [21]. This condition increases the likelihood of earlier HCC diagnosis through surveillance programmes for chronic HCV infection and cirrhosis [6,7]. Current survival probabilities are considerably higher for patients with HCC as compared with patients with ICC [17], reflecting a common international pattern [22].

Concerns have been expressed about some flaws in the current liver cancer policies. First, patients with HCC untreated with liver transplantation remain exposed to the mortality risk associated with the underlying chronic liver disease [23]. Second, the effect on survival resulting from increased monitoring of patients with HCV infection and cirrhosis may be subject to the lead-time bias [24]. Improvements in short-term survival do not necessarily translate into a higher probability of cure. It appears that the balancing of the benefits and harms of early detection may be particularly challenging in this population.

To obtain a better understanding of the impact of liver cancer control in Italy, we assessed the complete prevalence and indicators of cure (long-term survival) by histologic type.

## 2. Materials and Methods

### 2.1. Data

This study is part of a broader population-based research on the prevalence and cure after cancer, whose methods are described in detail elsewhere [25]. In brief, 31 cancer registries that met the following requirements were included: (1) availability of incidence data for at least nine consecutive years of registration before 2018; and (2) complete follow-up for vital status on 31 December 2018. Supplementary Table S1 shows the registries included, the years of registration available, the size of the resident population, the number of incident primary liver cancer cases contributed to the study, and the resulting average annual age-standardised (direct method, 2013 European standard population) incidence rate of total liver cancer per 100,000.

On 1 January 2018, the participating registries covered a total population of nearly 28,057,000, corresponding to 47% of the Italian population (with a coverage of 43% in northern-central Italy and 55% in southern Italy and the Islands). Their periods of registration varied from 1978 to 2017. Eligible patients were identified using the International Classification of Diseases for Oncology, 3rd edition, (ICD-O-3) [26] topography codes C22.0 and C22.1 with any ICD-O-3 morphology code except 8800-8969, 8971-8991, 9020, 9040-9044, 9050-9055, 9120-9133, 9140, 9150, 9170, 9180, 9220, 9231, 9240, 9251, 9260, 9364-9365, 9473, 9540, 9560-9571, 9580-9581, and 9590-9989 [17]. Specific ICD-O-3 morphology codes are based on histology verification.

Data from 111,006 primary liver cancer patients formed the basis of the study. Of these, 75,950 (68.4%) were males and 35,056 (31.6%) females. In the whole study population, the incidence rates of liver cancer were 25.9 per 100,000 males, 8.7 per 100,000 females and, combining both sexes, 7.4 per 100,000 for HCC (morphology codes 8170-8175 and 8970), 1.5 for ICC (8013, 8020, 8041, 8154, 8160-8162, 8180, 8240, 8246, 8249 and 8470) and 7.6 for ‘other liver cancer types’, which included cancers of unspecified type because of the lack of histologic verification (Supplementary Table S2).

### 2.2. Statistical Methods

The complete prevalence, the cure prevalence, the cure fraction (CF) and the life expectancy of fatal cases (LEF) were the study endpoints. A detailed description of the methods to estimate the complete prevalence and the indicators of cure can be found elsewhere [25]. In brief, the complete prevalence represents all previously diagnosed cancer survivors, regardless of the time elapsed since diagnosis. It was calculated as of 1 January 2018 by adjusting the prevalence observed in each registry with the completeness index method. The absolute number of prevalent cases in Italy for each cancer type was obtained as the sum of sex-, age-, and area-specific proportions calculated by pooling cancer registries, multiplied by the corresponding Italian population on 1 January 2018.

The cure prevalence is the proportion of prevalent patients who will not die of liver cancer. It was estimated for the total number of prevalent patients on 1 January 2018, and for the subgroups of prevalent patients who had already survived at least 5 and 10 years since diagnosis. The complement to one of the cure prevalences can be interpreted as the residual excess risk of death.

The CF is the proportion of newly diagnosed patients who have the same life expectancy as the general population and will not die of liver cancer. In other words, CF is the probability of experiencing no excess mortality. It was calculated using the mixture-cure model as the NS probability at the attained age of 100 years, which is the maximum age a person can be assumed to reach.

The LEF is the survival experienced by the 50th percentile of fatal cases or the median life expectancy of uncured patients, defined as those who will never experience the same mortality rate as the general population. It was calculated as the median distribution of the model-based NS obtained from the same Weibull models used for CF estimation.

For each cancer type and sex, the specific (i.e., independent) model which best fitted NS and conditional NS was applied.

Statistical analyses were performed using the SEER∗Stat, Version 8.4.0 (National Cancer Institute, Bethesda, MD, USA), SAS NLIN, version 9.4 (SAS Institute, Cary, NC, USA), and ComPrev, version 3.0.29 (National Cancer Institute, Bethesda, MD, USA) packages.

## 3. Results

Supplementary Table S3 shows the median age at diagnosis and the number of patients by sex, histologic type, and age group. Less than 2% of patients of both sexes were aged ≤44 years, and <10% were aged 45–54 years. The proportion aged ≥65 years was 69.0% among males and 84.2% among females. Males were younger irrespective of the histologic type. The column on the right shows the percent distribution (column percentages) by histologic type. ICC accounted for <10% of cases in both sexes. HCC was more frequent among males, but, notably, females were characterised by a higher proportion of ‘other liver cancer types’, which included unspecified types. As a related finding, females with ‘other liver cancer types’ were the oldest subgroup.

### 3.1. Complete Prevalence

Table 1 shows the complete prevalence of liver cancer on 1 January 2018 by histologic type and sex. The estimated total of 31,722 prevalent patients (both sexes combined) was equivalent to a proportion of 53 per 100,000 residents. Complete prevalence was mostly attributable to males (72.9%). Among men, the proportion of patients living after HCC was 62.7%, versus 52.6% in females.

**Figure 1:**
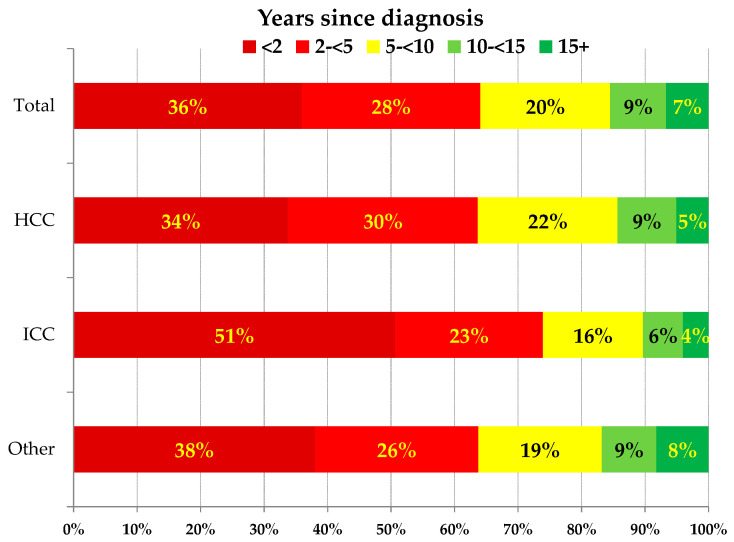
Percent distribution of liver cancer prevalence (both sexes combined) by histologic type and number of years since diagnosis in Italy (2018). HCC, hepatocellular carcinoma; ICC, intrahepatic cholangiocarcinoma. ‘Other’ liver cancer types include cancers of unspecified type because of the lack of histologic verification.

Figure 1 illustrates the percent distribution of prevalence (both sexes combined) by histologic type and number of years since diagnosis. Patients were distributed inversely proportional to the time elapsed. Overall, less than 20% of them had survived ≥10 years, with only a marginal difference being associated with the histologic type. Approximately two-thirds of total patients had been diagnosed less than 5 years earlier. The proportion of prevalent cases diagnosed with the disease in the last two years was significantly higher for ICC (51%) than for HCC (34%).

### 3.2. Cure Prevalence

Table 2 shows the cure prevalence (both sexes combined) by histologic type and number of years since diagnosis. Only 8886 liver cancer survivors (28% of all prevalent cases) will not die of liver cancer. This percentage was very low both among patients with HCC (24%) and ICC (40%), regardless of how long ago they were diagnosed. Even among subjects who had survived more than five years after diagnosis, the cure prevalence remained at 55.1% (total liver cancer cases) and 84.7% (ICC). Among patients diagnosed more than 10 years earlier, the percentage was 59.4% after HCC and 93.2% after ICC.

### 3.3. CF and LEF

Table 3 shows the CF and the LEF of males and females estimated for patients diagnosed in 2005 and in 2015 by histologic type and age at diagnosis. For both sexes and periods, the overall CF was below 10%. For males, the CF decreased roughly with increasing patient age regardless of the year of diagnosis. In the early period, no advantage for HCC versus ICC was observed, except for male patients aged <45 years. An increase over time was evident for both histologic types, and this change was more pronounced under the age of <45 years at diagnosis.

Females displayed a comparable pattern of results. The decreasing trend with increasing patient age, independent of the year of diagnosis, was confirmed. Under the age of 45 years, however, the CF was greater for HCC both in early and more recent cases. An increase over time occurred both for patients aged <45 years at diagnosis and for those aged 45–54 years. The LEF is shown in the column on the right of Table 3. In both sexes, the LEF was more than three times higher for HCC than for ICC. This occurred among early as well as recent cases. Uncured patients with HCC also showed an increase in LEF over time, particularly under the age of 55 years, which was definitely less for patients with ICC.

## 4. Discussion

The key results of this study may be summarised and commented on as follows. First, the overall CF of patients with HCC was below 10% in both sexes, irrespective of the period of diagnosis. The possibility of cure depends on the feasibility of either partial liver resection or liver transplantation. These primary curative options, in turn, are conditional on tumour down-staging through early detection [16] or neoadjuvant therapies [27]. Although partial resectability and liver transplantation have increased over time [28], and particularly so in Italy [29], their role in liver cancer control on a public health scale still appears to be, in our data, a small one.

Second, and closely related, no substantial advantage in CF was apparent for patients with HCC as contrasted with patients with ICC. The CF was greater only in the small age groups of 44 years or younger (<2% patients), where the requirements for regular ultrasound surveillance of HCV-infected and cirrhotic patients [13] and, consequently, for curative-intent hepatic resection and liver transplantation [30] are more frequent. The remaining patients with HCC were older and at a higher risk of being removed from surveillance and transplantation waitlists. By implication, their poor outcome impacted negatively on the overall CF. The management of older liver cancer patients has become a public health concern because of the ageing population and the increase in the risk of disease with advancing age. In Italy, however, incidence rates of HCC in the past two decades have decreased consistently for the age groups above 60 years [17].

Third, patients with HCC face an excess risk of death, which persists even decades after diagnosis. This explains the lower cure prevalence for patients with HCC as compared with patients with ICC, whose increased risk of death diminished more markedly over time since diagnosis.

Fourth, a more favourable finding was the clear-cut increase in CF observed when contrasting liver cancer cases centred in 2015 with those centred in 2005. The improvement was shared by both sexes and both histologic types and extended to patients aged 45–54 years. In some age and sex groups, the CF exceeded 20% and approached 30%.

Last, but important to note, the LEF was considerably greater for patients with HCC, and this was also the case for the latest years.

These findings complement and shed light on the information provided by national data on short-term survival. In Italy, short- and mid-term survival probabilities in the last two decades have improved and have been higher for patients with HCC [17]. The recent progress in CF for younger patients indicates that the improvement in survival from HCC has translated into decreased mortality. However, the growing LEF observed recently suggests that part of the prognostic improvement has not impacted the risk of dying. The reason is that most HCCs develop in a cirrhotic liver. Patients with cirrhosis remain at higher risk of death compared with the general population, especially in the presence of decompensation (ascites, portal hypertensive gastrointestinal bleeding, hepatic encephalopathy and jaundice) [23]. For patients with incurable disease, a prognostic improvement may result from better supportive care and from the lead-time bias [16,24]. The lead-time bias occurs when survival time appears longer only because the diagnosis of the disease is performed earlier through regular surveillance of HCV-infected and cirrhotic patients and not because the natural progression is decelerated and the date of death is postponed. This study supports the view that research on the epidemiologic biases associated with surveillance—which is very limited at present—should be expanded [24]. From this perspective, it is worth noting that the recently increasing use and the effectiveness of DAAs [31] in preventing the progression of chronic liver disease and controlling the incidence of HCC reduce the prevalence of subjects undergoing surveillance who are at risk of experiencing a lead-time-biased increase in survival from the disease. This is equivalent to saying that the temporal trend of this undesired side effect of surveillance for HCV infection is probably declining.

Summary statistics on the cure indicators for liver cancer have been previously reported from Italy [23,32], from a pool of European countries [33], and the U.S. [34], but none have presented results by histologic type. With respect to analytical studies, those published so far have considered clinical series of patients undergoing curative-intent hepatic resection. Although surgery often provides a clinical benefit, it is of utmost importance for the patients and the clinicians who care for them to determine whether hepatic resection is also effective in obtaining a cure. Contrasting the results of these studies with our data allows us to appreciate the magnitude of the impact of hepatic resection on the outcome of eligible patients. Regarding ICC, the CF has been reported to be near 10% [35]. For patients with HCC, the lowest figure has been around 17% [36] and the highest slightly above 40% [37,38]. In a systematic literature review with stringent study selection criteria, the range has been estimated between 31.8% and 40.5% with a time to cure (defined as time to reach a 5-year conditional NS > 95%) of 7–14 years [39].

Our approach was different. We considered an unselected population-based case series. This design, which is consistent with the intention-to-treat approach in clinical trials, was aimed at estimating the public health impact of liver cancer care, regardless of the extent to which patients were surgically treated [40]. Although the results underestimate the curability of liver cancer in the selected patients undergoing hepatic resection, applying the intention-to-treat principle yields a bias-free estimate of the effectiveness of liver cancer care under real-world conditions. From this perspective, this study may represent an important contribution to evaluating the impact of Italian national policies for HCC at the population level [41,42,43,44].

In this study, there are methodological issues to consider. A major one is the heterogeneity across registries in data quality and cancer registration practices. Comparisons between cure estimates after liver cancer are affected by the biases associated with the high proportion of death certificate only (DCO) registrations and the varying degree of completeness of the trace-back work. DCO cases, in general, are diagnosed shortly before death. Consequently, a high proportion of DCO cases with no or incomplete trace-back search, who are excluded from analysis, upwardly modify the estimated survival and cure probabilities [45,46]. In this regard, however, the completeness and accuracy of Italian data on the incidence and survival rates of liver cancer are deemed satisfactory [19,25].

A second limitation of this study is the considerable proportion of cases lacking histologic verification and, thus, of unspecified histologic type. This is quite a common problem worldwide [47]. In the past, registries were recommended not to indicate a histologic type without a documented tissue diagnosis. Due to the evolution of imaging, registration rules have been updated and, since 2023, they have allowed the assignment to a specific morphologic type of liver cancer based on imaging findings [48]. In parallel, improvements in imaging and the use of tumour markers have offered a partial solution to the problems of incorrect topographic attribution (intrahepatic, extrahepatic) of cancers of the bile ducts [49] and inaccurate assignment of primary cancer cases to metastases, and vice versa [22].

The limitations of cure models are discussed in detail elsewhere [22,25,33]. Particularly relevant to this study is the fact that the estimates are critical for cancers maintaining a long-term excess mortality risk, because the follow-up period available may not be sufficient to observe all deaths and, consequently, to observe the plateauing of the survival curve [23]. Additionally, NS may be affected by several biases, including both the lead time and the length biases, whereas the estimation of the time to cure is sensitive to the 5-year conditional NS threshold being chosen to indicate a low risk of recurrence or death. Cure fraction estimation is also sensitive to the assumptions and the statistical model used. However, cure at the population level is a reasonable and widely accepted hypothesis when the NS curves plateau and the excess mortality rate becomes negligible at some point within the follow-up interval [25].

## 5. Conclusions

Although limited by a considerable proportion of liver cancers of unspecified histologic type, the results of this study allow us to draw the following conclusions: (1) the CF of total liver cancer patients was <10%. (2) In the 2000s, no substantial advantages nor favourable trends in the CF were apparent for patients with HCC as compared to those with ICC, except for the small age groups of <45 years. (3) A clear-cut increase in CF of patients with HCC aged under 55 years occurred in the 2010s, with no changes for older patients. (4) This, however, was accompanied by an increase in the LEF. These findings suggest that the surveillance of HCV-infected and cirrhotic patients and advances in treatment have a two-fold effect: a true increase in the probability of cure of HCC and an increase in survival of fatal cases that is partially due to the lead-time bias. In summary, the cure indicators presented here may help to determine the long-term effects of therapeutic advances and patient monitoring in people with cirrhosis and HCV infection.

## Figures and Tables

**Table 1:** Complete prevalence of liver cancer on 1 January 2018. Number of patients and proportion per 100,000 residents by histologic type and sex in Italy.

Histologic Type	Males and Females			Males			Females		
	No.	%	Proportion (per 100,000)	No.	%	Proportion (per 100,000)	No.	%	Proportion (per 100,000)
Total	31,723		53	23,118		79	8605		28
HCC	19,015	59.9	32	14,493	62.7	50	4522	52.6	15
ICC	2308	7.3	4	1264	5.5	4	1044	12.1	3
Other	10,400	32.8	17	7361	31.8	25	3039	35.3	10

HCC, hepatocellular carcinoma; ICC, intrahepatic cholangiocarcinoma. ‘Other liver cancer types’ include cancers of unspecified type because of the lack of histologic verification. The complete prevalence includes all survivors, irrespective of the time interval since diagnosis.

**Table 2:** Cure prevalence of liver cancer on 1 January 2018. Number and proportion (%) of patients expected not to die as a result of cancer by histologic type and number of years since diagnosis in Italy.

Histologic Type	All Patients		Patients Diagnosed ≥ 5 Years Earlier		Patients Diagnosed ≥ 10 Years Earlier	
	No.	%	No.	%	No.	%
Total	8886	28.0	6277	55.1	3551	72.2
HCC	4488	23.6	2975	43.1	1623	59.4
ICC	930	40.3	510	84.7	222	93.2
Other	3468	33.3	2792	71.8	1706	87.6

HCC, hepatocellular carcinoma; ICC, intrahepatic cholangiocarcinoma. ‘Other liver cancer types’ include cancers of unspecified type because of the lack of histologic verification. The cure prevalence is the proportion of prevalent patients expected not to die as a result of cancer. The cure prevalence of patients diagnosed ≥5 and ≥10 years earlier is the probability of being cured conditional to surviving at least 5 or 10 years after diagnosis. The complement to 100% of cure prevalence can be interpreted as the residual risk of death.

**Table 3:** Cure fraction (%) of patients diagnosed with liver cancer and median life expectancy of fatal cases (months) by sex, year of diagnosis, histologic type, and age at diagnosis in Italy.

Sex	Year of Diagnosis	Histologic Type	Cure Fraction (%) by Age at Diagnosis (Years)						Life Expectancy of Fatal Cases (Months) All Ages
			All Ages	≤44	45–54	55–64	65–74	≥75	
Males	2005	Total	4	21	12	5	2	1	11.5
		HCC	4	13	7	5	3	3	19.8
		ICC	5	5	6	7	3	5	5.9
		Other	3	18	12	4	2	1	6.8
									
	2015	Total	8	27	20	12	6	3	16.9
		HCC	10	22	15	11	9	7	28.7
		ICC	8	23	11	8	5	11	7.2
		Other	7	21	21	10	5	3	9.9
									
Females	2005	Total	3	24	17	6	2	2	10.8
		HCC	4	19	11	7	4	3	21.1
		ICC	7	9	17	8	6	5	7.1
		Other	2	29	15	6	2	1	6.8
									
	2015	Total	7	33	27	12	6	5	15.2
		HCC	9	29	19	13	9	6	29.2
		ICC	11	16	30	8	9	9	8.5
		Other	5	43	26	10	4	3	9.7

HCC, hepatocellular carcinoma; ICC, intrahepatic cholangiocarcinoma. ‘Other liver cancer types’ include cancers of unspecified type because of the lack of histologic verification. The cure fraction is the proportion of newly diagnosed patients who have the same life expectancy as the general population, calculated using the mixture-cure model as the net survival probability at the attained age of 100 years, assumed to be the maximum age a person can reach. The life expectancy of fatal cases is the survival experienced by the 50th percentile of fatal cases or the median life expectancy of uncured patients, calculated as the median distribution of the model-based net survival estimated through Weibull distributions stratified by age.

## Data Availability

The data that support the findings of this study are available from the corresponding authors upon request.

## Supplementary Materials

The following supporting information can be downloaded at https://www.mdpi.com/article/10.3390/cancers18142318/s1. Supplementary Table S1. Selected characteristics of the 31 Italian local cancer registries participating in the study: years of registration, resident population, number of incident liver cancer cases contributed and average annual incidence rate of the disease. Supplementary Table S2. Average annual (2013–2017) liver cancer incidence rate by sex and histologic type in Italy. Supplementary Table S3. Median age at diagnosis and number of patients registered with liver cancer (1978–2017) by sex, histologic type and age group in Italy.

## Abbreviations

The following abbreviations are used in this manuscript:

CF	Cure Fraction
DAA	Direct-Acting Antiviral
DCO	Death Certificate Only
HBV	Hepatitis B Virus
HCC	Hepatocellular Carcinoma
HCV	Hepatitis C Virus
ICC	Intrahepatic Cholangiocarcinoma
ICD-O-3	International Classification of Disease for Oncology, 3rd edition
LEF	Life Expectancy of Fatal cases
NS	Net Survival
