# Supplementary material for: Prevalence and Cure of Primary Liver Cancer in Italy by Histologic Type
Source: Cancers (Basel). 2026 Jul 18;18(14):2318. doi: 10.3390/cancers18142318 (PMC13407252; doi:10.3390/cancers18142318)
Supplement: Supplementary file 1 [file cancers-18-02318-s001.zip › cancers-4357277-supplementary.pdf]

cancers

MDPI

Article

Prevalence and cure of primary liver cancer in Italy by histologic type

Lauro Bucchi, Stefano Ferretti, Federica Toffolutti, Ugo Fedeli, Silvia Mancini, Federica Zamagni, Fabiola Giudici, Laura Botta, Manuel Zorzi, Ettore Bidoli, Francesco Cuccaro, Cinzia Gasparotti, Adele Caldarella, Mario Fusco, Monica Lanzoni, Enrica Migliore, Antonella Puppo, Maria Michiara, Rossella Cavallo, Margherita Ferrante, Giuseppe Sampietro, Walter Mazzucco, Claudia Cirilli, Rosa Vattiato, Fabrizio Stracci, Linda Guarda, Federica Manzoni, William Mantovani, Giuseppe Cascone, Caterina Palmonari, Federica Targa, Lucia Mangone, Rocco Galasso, Antonio Porcheddu, Maria Teresa Pesce, Francesca Bella, Pietro Seghini, Anna Clara Fanetti, Pasquala Pinna, Stefano Guzzinati *, Luigino Dal Maso * and the AIRTUM Working Group

* Correspondence: dalmaso@cro.it (L.D.M.); Tel.: +39 0434 659 354; stefano.guzzinati@azero.veneto.it (S.G.); Tel.: +39 049 8778 130

Table of contents

Supplementary Table S1
Supplementary Table S2
Supplementary Table S3

Supplementary Table S1. Selected characteristics of the 31 Italian local cancer registries participating in the study: years of registration, resident population, number of incident liver cancer cases contributed and average annual incidence rate of the disease.

Cancer registry	Period of Registration	Population on 1 January 2018	No. of Incident Liver Cancer Cases in the Period of Registration	Average Annual (2013–2017) Liver Cancer Incidence Rate
Alto Adige – Sudtirol	1995–2017	527,750	1795	16.1
Basilicata	2005–2017	562,968	1711	19.1
Bergamo	2007–2017	1,111,035	3525	25.8
Brescia	1999–2017	1,162,241	6299	25.4
Caserta	2008–2016	915,730	1703	17.5
Catania-Messina-Enna	2003–2017	1,870,129	3888	13.1
Ferrara	1991–2017	348,030	2026	15.8
Firenze-Prato	1985-2016	1,269,331	5292	9.5
Friuli Venezia Giulia	1995–2017	1,211,155	6876	19.3
Genova	1993–2016	836,148	4383	12.1
Mantova-Cremona	1999–2016	763,482	2665	14.1
Modena	1988–2017	703,203	3410	15.2
Napoli	1996–2017	1,179,006	5287	31.6
Nuoro	2003–2015	209,100	812	14.7
Palermo	2003–2017	1,204,665	3417	16.4
Parma	1978–2017	449,858	4246	22.0
Pavia	2003–2017	545,810	2560	21.7
Piacenza	2006-2017	287,375	1035	23.7
Puglia	2006-2017	2,760,377	6493	17.0
Ragusa-Caltanissetta	1981–2017	587,799	2133	14.8
Reggio Emilia	1996–2017	533,649	1722	15.8
Romagna	1993–2017	1,126,342	3260	12.7
Salerno	1996–2017	1,091,434	3993	15.0
Sassari	1992–2015	328,581	1705	13.2
Siracusa	1999–2017	400,881	1373	18.7
Sondrio	1998–2017	181,025	993	23.3
Torino	1985–2015	860,793	4759	9.9
Trento	1995–2017	539,898	2449	20.4
Umbria	1994–2017	884,640	2944	10.1
Varese-Como	1990–2015	1,482,405	5257	8.9
Veneto	1990–2017	2,122,269	12,995	18.7
**Total**		**28,057,109**	**111,006**	**16.5**

* Incidence rates per 100,000 persons (both sexes combined) are age-standardised (direct method, 2013 European standard population).

Supplementary Table S2. Average annual (2013–2017) liver cancer incidence rate by sex and histologic type in Italy.

Histologic Type	Men	Women	Both Sexes
Total	25.9	8.7	16.5
HCC	12.5	3.1	7.4
ICC	2.0	1.2	1.5
Other	11.5	4.5	7.6

HCC, hepatocellular carcinoma; ICC, intrahepatic cholangiocarcinoma. 'Other' liver cancer types include cancers of unspecified type because of the lack of histologic verification. Incidence rates per 100,000 persons are age-standardised (direct method, 2013 European standard population).

Supplementary Table S3. Median age at diagnosis and number of patients registered with liver cancer (1978–2017) by sex, histologic type and age group in Italy.

Sex and Histologic Type	Median Age (Years)	No. (%) of Patients by Age (Years) at Diagnosis *					Total †
		≤44	45-54	55-64	65-74	≥75	
Men							
HCC	69	759 (2.1)	3159 (8.7)	8263 (22.7)	13,881 (38.1)	10,358 (28.4)	36,420 (48.0)
ICC	68	128 (3.2)	377 (9.6)	903 (22.9)	1485 (37.6)	1054 (26.7)	3947 (5.2)
Other	71	557 (1.6)	2673 (7.5)	6705 (18.8)	11,739 (33.0)	13,909 (39.1)	35,583 (46.9)
Subtotal	70	1444 (1.9)	6209 (8.2)	15,871 (20.9)	27,105 (35.7)	25,321 (33.3)	75,950
Women							
HCC	74	189 (1.5)	457 (3.6)	1637 (13.1)	4490 (35.8)	5771 (46.0)	12,544 (35.8)
ICC	71	103 (3.5)	246 (8.3)	565 (19.1)	985 (33.3)	1059 (35.8)	2958 (8.4)
Other	78	158 (0.8)	491 (2.5)	1710 (8.7)	4952 (25.3)	12,243 (62.6)	19,554 (55.8)
Subtotal	76	450 (1.3)	1194 (3.4)	3912 (11.2)	10,427 (29.7)	19,073 (54.4)	35,056
Total		1894 (1.7)	7403 (6.7)	19,783 (17.8)	37,532 (33.8)	44,394 (40.0)	111,006

HCC, hepatocellular carcinoma; ICC, intrahepatic cholangiocarcinoma. 'Other' liver cancer types include cancers of unspecified type because of the lack of histologic verification. * Numbers in parentheses are row percentages. † Numbers in parentheses are column percentages by sex.
